# Palm Temperature Differences after Static and Dynamic Load on High Bar

**DOI:** 10.3390/s21134497

**Published:** 2021-06-30

**Authors:** Karmen Šibanc, Ivan Čuk, Maja Pajek, Igor Pušnik

**Affiliations:** 1 Faculty of Sport, University of Ljubljana, SI-1000 Ljubljana, Slovenia; karmen.sibanc@fsp.uni-lj.si (K.Š.); ivan.cuk@fsp.uni-lj.si (I.Č.); maja.pajek@fsp.uni-lj.si (M.P.); 2 Faculty of Electrical Engineering, University of Ljubljana, SI-1000 Ljubljana, Slovenia

**Keywords:** palm temperature, thermal imaging, hang, swing in hang

## Abstract

Thermal imaging is used in various fields of industry and research to measure temperature and its possible differences. Since there is a lack of research and literature on palm temperatures and prevention of blisters on hands, our question was how palm temperature differs in human hands after different loads (Hang and Swing in Hang) for 30 s on a high bar. Thirty-eight students from the Faculty of Sport at the University of Ljubljana were measured with a high-quality thermal imaging camera. Palm temperatures were measured before the load was applied, immediately after and every 30 s for a period of 5 min after the load. Each hand was divided into nine different regions of interest (ROIs). Mean (XA), standard deviation (SD), maximum and minimum, and number of pixels were calculated. We found that there was no difference between the left and right hand. The temperature right after the load was applied decreased significantly for both loads and then increased above the level before the load was applied. After the static load, the temperature reached a constant higher level after 3 min. After the dynamic load, the temperatures continued to increase throughout the measurement period. Further investigation is needed to determine the time period in which the hand temperature reaches the temperature before the load is applied.

## 1. Introduction

The use of thermal imaging has increased in recent decades. It is now used in various fields of research (non-destructive material testing, aerial thermography, high-speed thermography, inspection of mechanical components, thermography in material testing, thermography in medicine and veterinary medicine, micro-thermography, infrared cameras for plant inspections, infrared cameras for security, sport science) and industry (aerospace industry, automotive industry, chemical industry, electronics and electrical industry, and metallurgy) [1].

Infrared thermography has been successfully used in medicine to diagnose breast cancer, diabetic neuropathy, and peripheral vascular disorders, as well as to detect problems in gynecology, kidney transplants, dermatology and chronic wound treatment [2], the heart, neonatal physiology, fever screening, and brain imaging [3,4], and dentistry [5]. In 2017, a review on the application of medical infrared thermal imaging in hands was conducted [6]. It considered 146 articles that reported on several different fields of application and presented thermal imaging as a potentially promising tool for additional non-invasive diagnostics.

In the majority of healthy subjects [7], it was observed that the temperature of the hands, feet and facial regions increased after immobilization, which can be explained by the fact that immobilization is usually followed by relaxation and blood redistribution, resulting in an increase in skin surface temperature. In addition, spontaneous oscillations have been observed in the temperature profiles of the hands and feet due to changes in the sympathetic system and blood flow. Oscillations that have a period of less than 4 min are associated with the capillaries, while those with higher periods are due to the arterio-venous anastomosis. In another study [8], cold stress was applied by placing the left hand on a cold metal surface (approximately 13 °C) and observing the temperature dynamics on both hands using infrared thermography. It was observed that after a steady-state period of 85 s, the temperature on the stimulated hand decreased, while the temperature on the other hand increased over the next 90 s. The temperature on the stimulated hand decreased and the temperature on the other hand increased. This phenomenon is attributed to the fact that the thermoregulatory mechanism responds in such a way that the core temperature remains undisturbed.

Thermal imaging is also part of research on wellbeing and in different fields of sport science that are trying to develop optimal training and results and health [9,10,11]. Movement of the hand consists of two basic patterns, which are termed the precision grip and the power grip [12]. In the field of sports, there are quite a few activities where the hands form a power grip and disciplines where the body is hanging (artistic gymnastics, sport climbing, fitness, Crossfit, etc.). Most of these disciplines and activities take place in a room temperature environment, but there is a lack of data on how applying palm load directly affects the palm and hand. In the power grip, extrinsic muscles play a key role in the movement by providing the major force [12].

Artistic gymnastics is the oldest organized sport. In competitions, gymnasts perform a routine consisting of various elements and the connection of such elements. Each element is defined first by the position of the gymnast according to an apparatus, secondly, by a grip or the touch of a gymnast on an apparatus, and then, by the description of the movement to the final position of an element. Routines on a high bar, rings, parallel bars, uneven bars and the pommel horse usually last between 20 and 50 s, with an average of 30 s [13]. Skin injuries occur on artistic gymnastics’ apparatus, where gymnasts’ palms are exposed to frictional forces [14]. Frictional forces combined with pressure cause shear forces on the skin, which affect blood circulation [15,16], or more precisely, microcirculation [17], and repetitive loads and friction have effects on temperature elevation, which can cause discomfort and subsequently, skin injuries [18]. Thinner skin is more likely to blister or injure, while thicker skin requires a longer duration of stress and higher friction for injury [19]. Investigations on microcirculation in both the dorsal and palmar sides of the human fingertip have revealed anatomical and microstructural differences between the sides [17]. On the palmar side, capillary loops are arranged in rows that follow the ridges of the fingerprint, whereas the capillary loops on the dorsal side are more randomly distributed, changing orientation and direction due to the different parts of the fingernail [7].

The temperature dynamics of the surface of the human body at rest under thermoneutral conditions are determined mainly by peripheral blood flow controlled by the degree of the vasoconstriction, which is controlled almost entirely by the sympathetic nervous system in response [20]. However, blanching of the skin after the application of pressure (as happens in palms during a long swing on rings) is a well-known phenomenon, although the associated changes in skin temperature are not well investigated. The response of skin temperature to exercise has been studied [21], with a steady load leading to a constant decrease in finger temperature followed by rewarming of the hands, reflecting the dominance and balance of thermoregulatory reflexes and hemodynamics in the later phase of exercise. Graded load exercise results in a constant decrease in finger temperature due to the constant dominant vasoconstrictor response. Local cooling has been used in rehabilitation or between intense exercise sessions to reduce swelling and inflammation, and the effect of hand cooling on fatigue during high-intensity bench press has been investigated [22], but we found very few studies related to applying load or pressure to palm or handling objects at room temperature in a given time range. Bennett et al. [23] studied hand temperature after pressure for 5 min and discovered an initial cooling of the hand right after load application, and the application of heavy pressure resulted in a hotter hand than the application of light pressure.

When performing simple elements on uneven bars, palm temperature differences were compared with and without the use of magnesium carbonate [24]. The palm temperature increased with the use of magnesium carbonate, while the temperature remained constant without the use of magnesium carbonate. The short-term effects of applying load on palm temperature with different shapes of gymnastic rings were compared [14], and statistical differences were found in the decrease of palm temperature after the load was applied, depending on the different shapes of rings. As one of the possible explanations for the lowering of palm temperature, the researchers point to the movement in the hanging position on rings, since the palm as the contact surface is high above the level of the heart at the reachable height, which causes a disturbance in blood flow to the hand. At rest and under thermoneutral conditions, the temperature dynamics of the human body surface is mainly determined by peripheral blood flow [25].

In our research, we were interested in the differences between the static and dynamic load applications, where the friction force is different.

In recent research [14], the thermograms showed a different temperature distribution in the area of the hands, so, we focused on more regions of interest (ROI) according to the load. Healthy skin temperatures are supposed to be regarded as symmetrically distributed [26], however, according to the demonstrated asymmetries in gymnastics [27,28] and a study that showed left-right asymmetry in hand temperature after cold stress [29], we also checked whether there are asymmetries between the left and right hands after the load was applied. Since temperature recovery period is not well investigated, according to Gulyaev et al.’s research [7], where temperatures returned to their level before occlusion after 2–3 min, the selected period for our measurement was 5 min, even though Bennett et al.’s study [23] showed some different patterns after a 10 min recovery period.

From the above studies [3,4,7,8,14], it was found that in grasping during the execution after the loads and their amplitude, the differences in the temperature of the skin of the palm were very different, and in long-term hanging, it was found that the temperature of the palm decreased. Therefore, we wanted to find out what happens in the case of static (Hang) and dynamic loads (Swing in Hang, denoted as “Swing” in the following text) when hanging on the high bar for a duration of 30 s, which is the average duration of the exercise performance. When hanging, the entire body is under contact with an apparatus.

## 2. Materials and Methods

The subjects were 38 (27 women and 11 men) healthy students from the Faculty of Sport, University of Ljubljana. The average age was 22.1 years (±2.9), height 1.73 m (±0.10), and body weight 72.2 kg (±12.5). All included subjects gave written informed consent and the Declaration of Helsinki was followed. Ethical approval was obtained from the Ethical Commission of the University of Ljubljana’s Faculty of Sport (12_2018).

The authors developed the measurement protocol according to our hypotheses:

**Hypothesis** **1.** *Palm temperature decreases after application of the static load in hang.*

**Hypothesis** **2.** *Palm temperature increases after application of the dynamic load in swing while in hang.*

**Figure 1:**
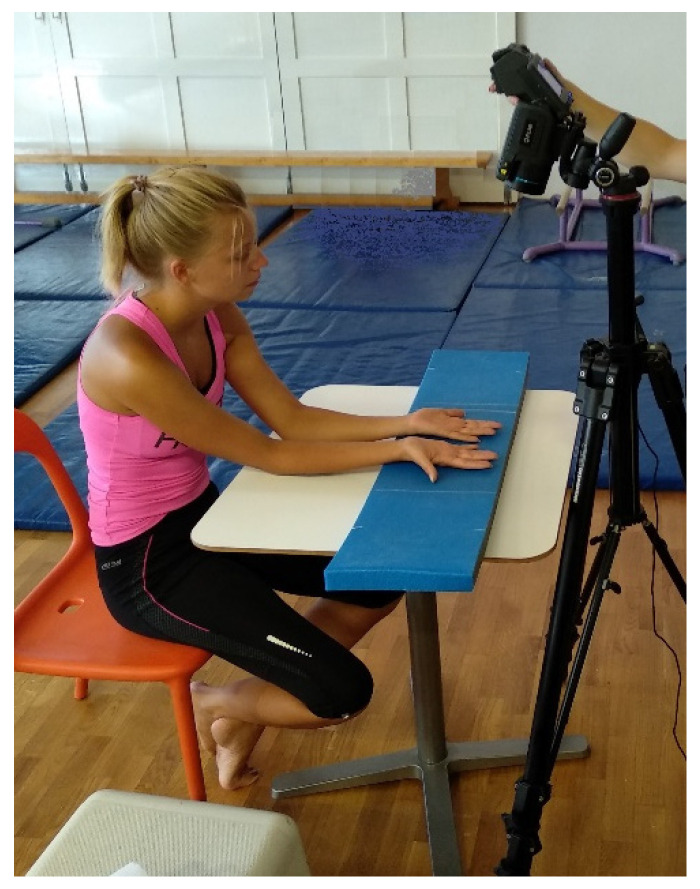
Camera setting while taking thermal images.

**Figure 2:**
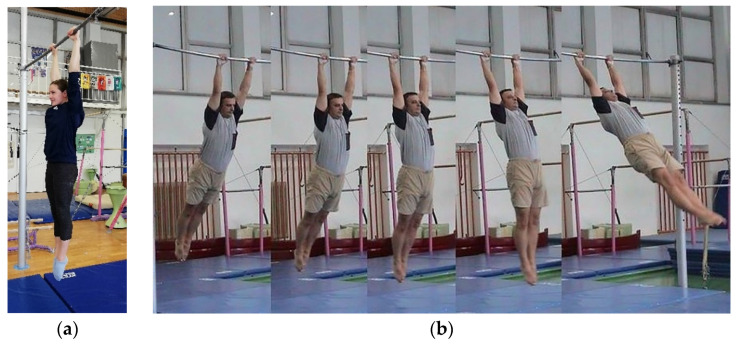
(**a**) Hang and (**b**) Swing.

Hand temperatures of the subjects were measured before the load was applied, immediately after the load, and then for 5 min at 30 s intervals (12 thermograms per subject per load). The thermography setting is shown in Figure 1: the subject is in a seated position, hands resting on the table with fingers spaced at approximately the level of the heart and on an additional insulating surface to prevent heat loss into the table. Subjects were required to perform two tasks on the high bar (steel, ø = 28 mm); 30 s of still hanging (Figure 2a) and 30 s of swinging while hanging (Figure 2b) with a fixed swing range (approximately 30 degrees from the vertical position). Subjects were evenly and randomly assigned the task that they performed first. The handgrip for both tasks was a cylindrical power grip. The measurements took place in the gymnastics hall of the Faculty of Sport, University of Ljubljana. The temperature in the hall was 23 °C and the relative humidity was 40 %. All subjects were in the hall for acclimatization 15 min before the start of the first measurement. They were asked not to drink alcohol or smoke for 24 h, not to consume caffeine for 12 h, and not to use creams or lotions on their hands for 12 h before the measurements [30]. The skin on their hands was not visibly damaged.

A high-quality thermal imaging camera (FLIR T650sc FLIR Systems, Wilsonville, OR, USA) was used in the study. The detector type was an uncooled microbolometer with the FPA, operating in the spectral range from 7.5 μm to 14 μm, noise equivalent temperature difference NETD < 30 mK, and a specified accuracy of ±1% of reading or 1 °C in the temperature range from 5 °C to 120 °C, at ambient temperatures 10 °C to 35 °C. The camera was calibrated prior to the research measurements in the LMK-Laboratory of Metrology and Quality at the Faculty of Electrical Engineering, University of Ljubljana (LMK is the holder of the Slovenian national standard for thermodynamic temperature. As a national laboratory for temperature and relative humidity and as an accredited calibration laboratory, it has CMCs-calibration measurement capabilities that are among the best in Europe and worldwide in the respective fields of measurement, which confirms its accuracy. The camera has a wide-angle 45° lens (f = 13.1 mm) with the field of view (FOV) of 45° × 34° mm, spatial resolution of 1.23 mrad (IFOV), continuous zoom (8×) and a minimum focus distance of 15 cm. The emissivity value can be set in steps of 0.01 from 0.10 to 1.00. Measurements were corrected for the reflected temperature, optics transmission, atmospheric transmission and external optics. Image analysis in the associated software environment (ResearchIR Max by (FLIR Systems, Wilsonville, OR, USA) enabled the analysis of different ROIs, e.g., spots, areas, automatic hot/cold point detection, difference of temperature, isotherms, line profiles, alarms, temporal temperature dependence, etc. The resolution of the camera is 640 × 480 pixels, which amounts to 307,200-pixel temperatures in a single thermogram. This high resolution is important for a comprehensive analysis of the selected ROIs [31].

**Figure 3:**
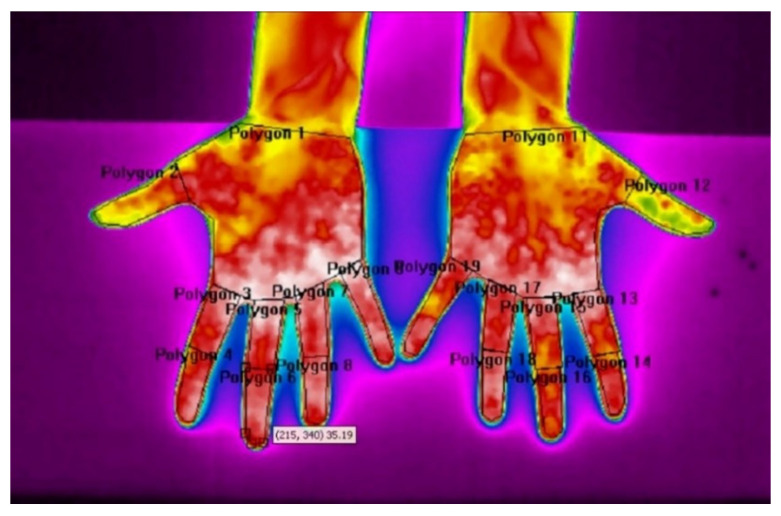
Thermal image and ROI on hands.

We divided each hand into nine ROIs (polygons), as shown in Figure 3, using the ResearchIR application: Palm, Thumb, Index Finger Proximal Phalanx, Index Finger Distal Phalanges, Middle Finger Proximal Phalanx, Middle Finger Distal Phalanges, Ring Finger Proximal Phalanx, Ring Finger Distal Phalanges and Little Finger. Table 1 contains the ROIs shown in Figure 3 and the abbreviations used in the following text for each ROI. A very important point in the analysis of thermal images is the drawing of ROIs, taking into account that the boundary of the ROI must be at least 7 pixels away from the edge of the observed surface to avoid the influence of the size-of source effect [32].

Data from ResearchIR were exported to Excel for Windows 10 (Microsoft Corporation, Redmond, WA, United States) for each ROI including mean (XA), standard deviation (SD), maximum and minimum, and number of pixels. In Excel, we calculated two new variables. The first one represents the sum of temperature differences of the section before the task until the end of the 5th minute using the formula Σ(T_n_ − T_n_ + 1), where n represents the time series. The second variable was calculated as temperature differences from the first to the last measurement using the formula T_1_ − T_n_ + 1, where n represents the time series. Statistical analyses were performed in SPSS 25.0 (IBM corp.).The values of the Kolmogorov—Smirnov test, means (XA), standard deviations (SD), standard errors (SE) were calculated for each sector variable and the pairwise Student’s t-test was used (to compare the temperature difference between Hang and Swing, between the left and right sectors, and between the time series in each task). The figures and graphs were created using Excel software.

## 3. Results

According to the Kolmogorov–Smirnov test, variables were not normally distributed.

No significant difference was observed between men and women, which was also found in other studies [33]. Since there was no significant difference between the left and the right hand, the results are presented for the left hand.

For all ROIs, there was a significant difference (*p* < 0.05) between the temperatures in Hang and Swing. The sum temperature difference in 330 s (the total measurement time including load in the first 30 s) for the Left Hand from the ROI is shown in Table 2.

All values were higher for Swing than for Hang, with the minimum difference always being Palm and the maximum difference always being Little Finger. We can also see that there was a difference between the proximal phalanx and distal phalanges for the Index, Middle and Ring Finger. The average of mean temperatures (XA) in Swing was higher than the average of mean temperatures in Hang, ranging from the minimum 0.63 °C (for Palm) to the maximum 1.83 °C (for Little Finger), the minimum SD was 0.34 °C for Palm and the maximum was 1.14 °C for Little Finger, the minimum SE was 0.06 °C for Palm and the maximum was 0.18 °C for Little Finger.

Table 3 shows the significant difference in temperature by time and region before and after applying load, which was applied for each 30 s during a period of 5 min, where “+” means a significant difference in temperature by *t*-test (*p* < 0.05). In Hang, the difference in temperature every 30 s after the application of load was significant for all ROIs up to 150 s, for Palm up to 180 s, and for all Proximal Phalanx up to 210 s.

According to Swing, the temperature difference was significant for all ROIs until 180 s after applying the load, reached a plateau at 210 s where the difference was not significant, the difference was again significant for all ROIs after 240 s and then again for Proximal Phalanx and Palm in the last minute of the measurement.

**Figure 4:**
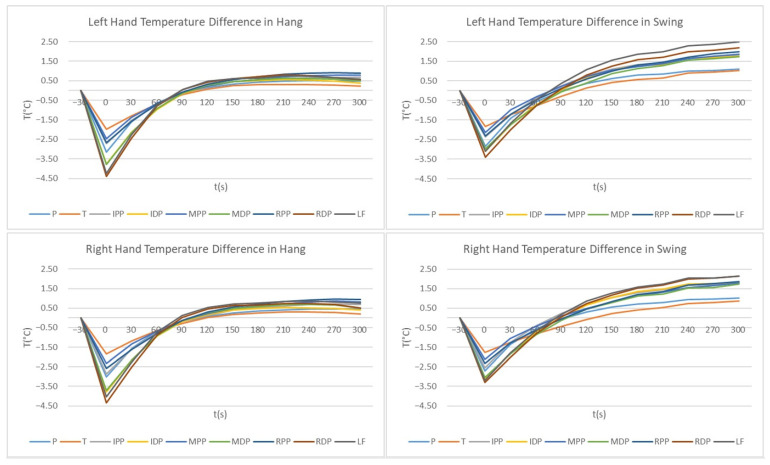
Right- and Left-Hand temperature difference in Hang and Swing. Both charts on the left side of the figure present temperature differences for Hang and the charts on the right side of the figure present temperature differences for Swing. The upper two charts present the left hand and the lower two charts present the right hand.

**Figure 5:**
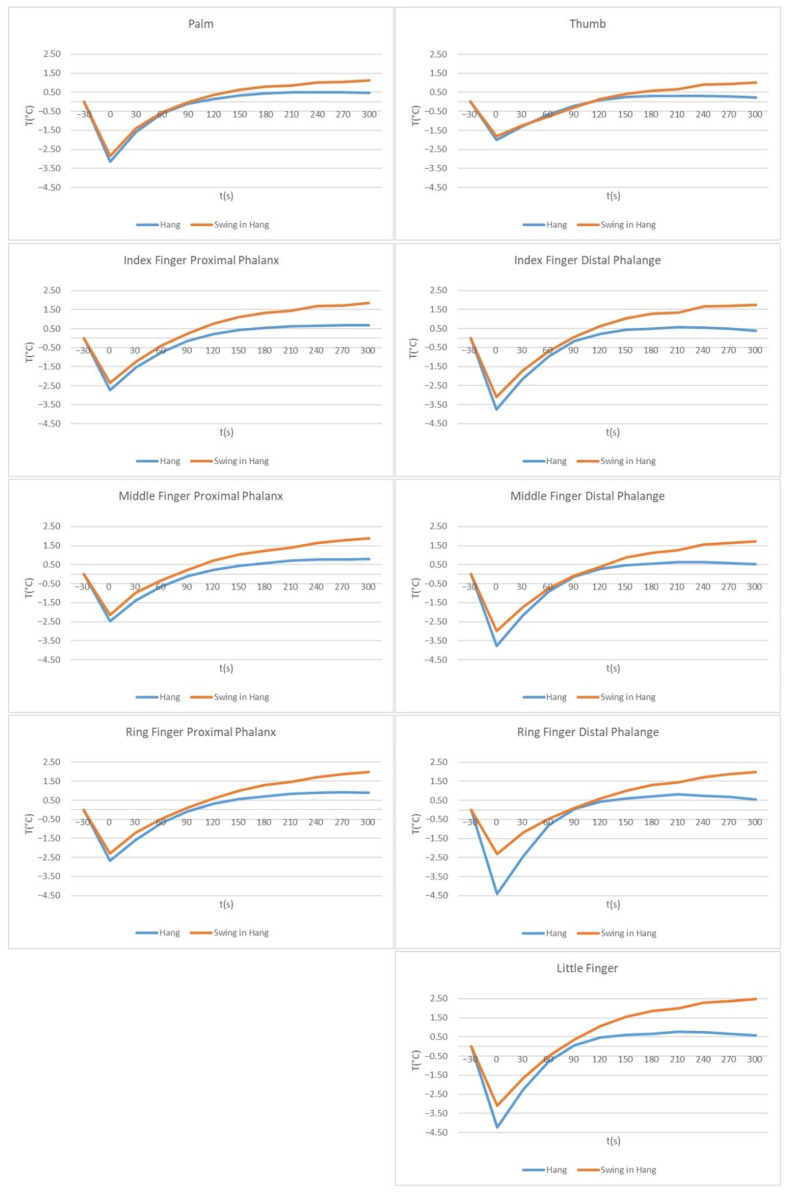
Left-Hand temperature difference in Hang and in Swing for different ROIs. Charts on the left side of the figure present temperature values for Palm and all three Proximal Phalanx; charts on the right present values for the Thumb, all three Distal Phalanges and Little Finger. The values for Index, Middle and Ring Finger Phalanx/Phalanges are in line for each finger.

The mean temperature differences with respect to the time course are shown in Figure 4 and Figure 5.

The temperature differences between Hang and Swing are shown in Table 4. Immediately after the application of the load, a significant difference was observed for the Palm, Little Finger and all fingers of the distal phalanges. For the Index Finger Proximal Phalanx the temperature differences were significant from 60 s to the end of the 5 min period. Up to 210 s, the temperature distribution was similar for each ROI and the difference was not significant. For all ROIs, from 240 s onwards, there was a significant difference, as the temperatures of all ROIs were higher after Swing than after Hang.

Figure 4 shows the mean temperature differences for Hang and Swing for the left and right hand for all the measured ROIs in the hands.

There was hardly any difference between the left and the right hand for both loads, while a significant difference between the loads can be seen in Figure 5. For both loads, for ROIs where the temperature decrease was larger, the temperature increase was larger according to the temperature before the load. The temperature decrease for Hang was larger than for Swing (after Hang, the temperature decrease was 1.99–4.39 °C and after swing, it was 1.83–3.40 °C). After both loads, Thumb had the smallest temperature difference. After Hang, the temperature values of the ROIs decreased immediately after the load by −1.99 °C (Thumb) to −4.39 °C (Ring Finger Distal Phalanges) corresponding to the temperatures before the load. Three hundred seconds after Hang, the temperature of the ROIs increased to larger values than before applying the load for 0.22 °C (Thumb) to 0.90 °C (Ring Finger Proximal Phalanx). After Swing, the temperature of the ROIs decreased to less than after Hang, from −1.83 °C (Thumb) to −3.40 °C (Ring Finger Distal Phalanges), corresponding to pre-load temperatures. Three hundred seconds after the Swing, temperatures increased by 1.02 °C (Thumb) to 2.49 °C (Little Finger), corresponding to pre-load temperatures.

In Hang, the rise in temperature above the initial value (before the load was applied) was very similar for all ROIs, appearing between 90 and 120 s after the load, while after Swing, the temperatures of the ROIs reached their initial values between 75 s and 110 s after the load. As shown in Table 3, the temperature changes after Hang reached a plateau about 150 s to 180 s after the load, which can also be seen clearly from the curves in Figure 4 and Figure 5. After the Swing (according to Figure 4 and Figure 5 and Table 3), the plateau can be seen at 210 s, but the temperatures still increased significantly after 240 s.

Figure 5 shows the temperature difference between Hang and Swing for each ROI separately. The smallest difference between Hang and Swing was for the Palm. The Thumb in this study showed the smallest temperature difference in general—in addition to small temperature differences between loads (up to 120 s after load application), it also showed the smallest temperature decrease after load. A difference was observed between the proximal phalanxes and distal phalanges. For all distal phalanges and the Little Finger, the temperature differences between Hang and Swing were larger than for the proximal phalanxes (and Palm and Thumb), as mentioned above. The greater difference between Hang and Swing was in the Ring’s Finger Distal Phalange immediately after load application, and in the Little Finger 90 s after load application. In the index and middle fingers, the temperature decrease was much larger in distal phalanges. In the ring finger, the temperature difference between the phalanx and phalange after Swing was not very large, while in Hang, the temperature decrease after load application was larger in the Distal Phalange than in the Ring Finger’s Proximal Phalanx.

## 4. Discussion

Despite the demonstrated asymmetries in artistic gymnastics [27,28], the temperature of the hands in a static position is symmetrical in healthy subjects [34], and the temperature difference and distribution between the left and the right hand in our study before and after the load application appeared to be symmetrical. The loads included in this study, Hang and Swing, are symmetrical positions/movements (compared to the aforementioned studies on gymnastics) and did not include movements such as rotations or twists around the longitudinal or transverse axis, hanging on one hand, etc. This means that possible body asymmetries had no influence on the temperature differences of the hands after load application.

The temperature difference was higher in all ROIs under dynamic load than under static load, the *t*-test proves a significant difference between the loads for the whole period of our measurement, with the biggest difference being on the distal phalanges of the ring and index finger, as well as the Little Finger. This could be related to a previous study [7], where the authors showed that the response to the hands starts at the fingertips and progresses in a wave-like manner to the upper parts of the hand. In their study, they examined hand temperature after immobilization, which was similar to our study but without the load. Adding the load indicates greater forces on the skin, considering gravity and grip during the Swing, the distal phalanges bear most of the body weight in the second part of the swing where the whole body (except the palms) was in front of the bar. This is also evident in the comparison of temperature differences after the Hang and the Swing (Table 4). Significant differences immediately after load application were found on the Palm, Little Finger and distal phalanges of all three fingers. Thirty seconds after load application the significant difference was at the proximal phalanxes of the middle and ring fingers and the Little Finger, showing the different responses of the palms to the temperature distribution and thermoregulation mechanism. Significant temperature differences between Hang and Swing were observed in all ROIs 240 s after loading, but we cannot forget that significant temperature differences were prominent for the Index Finger Proximal Phalanx and Little finger. As personally experienced by the authors, the index finger seemed to be the most loaded during Hang and Swing. Further research on this topic is needed. Research on rings [14] found that the reasons for lower palm skin temperature (immediately after load application) may be related to the position of the hands above the heart and the shape of the rings. When the fist is formed to grip, it forms an outward bowed shape, which would mean that the Index and the Little Finger are subjected to higher pressure and gripping force when hanging on a bar; this is also due to gravitational force.

As shown in Table 3, the temperature differences were significant every 30 s. For a longer period, the differences were significant for the Palm and proximal phalanxes; the temperatures of the Thumb and distal parts of the Palm reached a certain level where the temperature was higher than before the load application and no longer changed significantly. This is similar for both loads, but happened earlier in Hang than in Swing. In Hang, the temperatures reached a new constant level after 3 or 4 min, which we did not observe after Swing.

The Thumb had the smallest temperature difference. According to the cylindrical power grip, where the Thumb is oriented differently compared to digits 2–5, it is a key anatomical feature of the hand that allows for this opposite orientation and a much stronger grip [35]. The Thumb was the only finger that was under the bar and was not affected by gravity like the other fingers. Skin contact with the bar and the thumb was mostly on the inner side of the thumb and the angle of our settings was not ideal for sensing the thumb’s temperature.

As shown in Figure 4 and Figure 5, for all measurements, we see the temperature decrease immediately after the load was applied. After the static load, the temperature decrease was greater than after the dynamic load, but then, the temperatures reached a higher constant level (corresponding to the pre-load temperatures) after about 180 s, remained at this level until 240 or 270 s, and then the temperatures decreased again slightly (except for the proximal parts of the index and middle fingers). Immediately after dynamic load application, temperatures decreased (lower than after static load), followed by a mostly similar increase in temperature up to 90 s. The index and middle finger temperatures were similar. Subsequently, temperatures of all ROIs reached higher values than after the static load (see Figure 5), and temperatures continued to increase until 300 s after load application. Longer measurements would be necessary to determine the time at which temperatures reached pre-load application values. Repetitions of exercise routines are necessary in gymnastics training [13], and constant loading of the palms and a constant increase in palm temperature could lead to blisters on the palms. Blisters are the most common injury of the palm. However, they are not counted among the typical injuries because they are not treated in medical centers [36,37]. With proper rest during training and competition, palm injuries could be reduced.

One of the possible explanations for a decrease in palm temperature could be the change in the blood flow in the palm while executing elements in the hanging position [14]. Measurement of skin blood flow is usually accomplished by laser Doppler flowmetry or venous occlusion plethysmography This method offers the advantages of high temporal resolution (measurements can be taken continuously) and specificity to the cutaneous microcirculation. It has already been found that when the fist was clenched the blood flow had high resistance and after releasing (after 3 min) the blood flow had low resistance. This response is mainly the consequence of ischemia of the whole palm, which occurs with clenching of the fist [38]. To evaluate this hypothesis and better investigation we suggest that future studies should include measurements of mean volume flow through the radial artery.

## 5. Conclusions

In artistic gymnastics, palm loads are part of training and competition on all apparatus. Especially on the bars (high bar, parallel bars and uneven bars) and on the rings, the hanging position is performed, where the risk of palm injury due to palm load application is high. With this study, we can conclude that in Hang (static load) and Swing (dynamic load), the temperature distribution for the left and right hand is symmetrical. In both static and dynamic load application, temperatures decreased significantly by about 3 °C (±1.2 °C) immediately after the load application and then began to increase, reaching higher values than before the load was applied. Consequently, our first hypothesis was accepted and the second hypothesis was rejected. After the static load was applied, the temperature decrease was significantly larger than after the dynamic load (by 0.5 °C on average) and after about 210 s, when the highest temperature values were reached (between 0.32 and 0.90 °C higher than before the load was applied), it slowly started to decrease. After application of the dynamic load, the temperature decrease was lower than after application of the static load, but then continued to increase over the entire period of measurement, and after 5 min it was 1.0–2.5 °C higher than before the load was applied.

## 6. Strength and Limitations

Because of the large database, Tables with all values were not included.

With this study, we started to question the efficiency of time of rest for possible skin injury prevention. According to our results, further investigations should be conducted to obtain more information about the time for skin temperature to reach its value before the load. Thermography, as a non-invasive and non-radiating analysis tool has not been used to answer this question yet. In further research, it has to be determined when skin is ready for the next load without the risk of potential skin damage, maybe even calculating and representing a cooling curve for hands might be possible.

The values for the thumb are correct for this angle of measurement, but since the main contact with apparatus is on the inner side of the thumb, measuring separately and from a different angle would give different temperatures. To get more precise data, the thumb should be measured from a different angle.

When we observe low temperature differences (in the order of a tenth of a degree Celsius), it is not enough to rely on any non-contact temperature device because low-cost radiation thermometers and thermal imagers are not capable of accurate or consistent measurement of temperature differences. Therefore, the use of an accurate (calibrated) non-contact temperature device is necessary.

## Figures and Tables

**Table 1:** ROIs on each hands.

Hand	Polygon	ROI	Abbreviation
Right Hand	Polygon 1	Palm	P-R
	Polygon 2	Thumb	T-R
	Polygon 3	Index Finger Proximal Phalanx	IPP-R
	Polygon 4	Index Finger Distal Phalanges	IDP-R
	Polygon 5	Middle Finger Proximal Phalanx	MPP-R
	Polygon 6	Middle Finger Distal Phalanges	MDP-R
	Polygon 7	Ring Finger Proximal Phalanx	RPP-R
	Polygon 8	Ring Finger Distal Phalanges	RDP-R
	Polygon 9	Little Finger	LF-R
Left Hand	Polygon 11	Palm	P-L
	Polygon 12	Thumb	T-L
	Polygon 13	Index Finger Proximal Phalanx	IPP-L
	Polygon 14	Index Finger Distal Phalanges	IDP-L
	Polygon 15	Middle Finger Proximal Phalanx	MPP-L
	Polygon 16	Middle Finger Distal Phalanges	MDP-L
	Polygon 17	Ring Finger Proximal Phalanx	RPP-L
	Polygon 18	Ring Finger Distal Phalanges	RDP-L
	Polygon 19	Little Finger	LF-L

**Table 2:** Sum temperature difference for Hang and Swing in 330 s for Left Hand by ROI.

Variable	XA/°C	XA_H_-XA_S_/°C	SD/°C	SE/°C	*p* (*t*-Test)
Hang P ^a^-L	0.46	0.63	0.70	0.11	0.003
Swing P ^a^-L	1.09		1.04	0.17	
Hang T ^b^-L	0.19	0.83	0.92	0.15	0.004
Swing T ^b^-L	1.02		1.43	0.23	
Hang IPP ^c^-L	0.66	1.16	1.09	0.18	0.000
Swing IPP ^c^-L	1.83		1.55	0.25	
Hang IDP ^d^-L	0.33	1.42	1.21	0.20	0.000
Swing IDP ^d^-L	1.75		1.89	0.31	
Hang MPP ^e^-L	0.77	1.08	0.93	0.15	0.001
Swing MPP ^e^-L	1.85		1.54	0.25	
Hang MDP ^f^-L	0.49	1.23	1.13	0.18	0.001
Swing MDP ^f^-L	1.71		1.86	0.30	
Hang RPP ^g^-L	0.89	1.06	1.03	0.17	0.005
Swing RPP ^g^-L	1.95		1.76	0.28	
Hang RDP ^h^-L	0.52	1.63	1.32	0.21	0.001
Swing RDP ^h^-L	2.15		2.27	0.37	
Hang LF ^i^-L	0.57	1.84	1.13	0.18	0.000
Swing LF ^i^-L	2.41		2.27	0.37	

*Note:*^a^ P = Palm; ^b^ T = Thumb; ^c^ IPP = Index Finger Proximal Phalanx; ^d^ IDP = Index Finger Distal Phalanges; ^e^ MPP = Medial Finger Proximal Phalanx; ^f^ MDP = Medial Finger Distal Phalanges; ^g^ RPP = Ring Finger Proximal Phalanx; ^h^ RDP = Ring Finger Distal Phalanges; ^i^ LF = Little Finger.

**Table 3:** Difference in temperature by time and region (where + means significant difference by *t*-test (*p* < 0.05)) for Hang and Swing for Left Hand.

Variable	−30/s	0/s	30/s	60/s	90/s	120/s	150/s	180/s	210/s	240/s	270/s	300/s
Hang P ^a^-L		+	+	+	+	+	+	+				
Swing P ^a^-L		+	+	+	+	+	+					
Hang T ^b^-L		+	+	+	+	+	+	+	+			
Swing T ^b^-L		+	+	+	+	+	+					
Hang IPP ^c^-L		+	+	+	+	+	+	+	+			
Swing IPP ^c^-L		+	+	+	+	+	+					
Hang IDP ^d^-L		+	+	+	+	+	+	+	+			
Swing IDP ^d^-L		+	+	+	+	+	+					
Hang MPP ^e^-L		+	+	+	+	+	+					
Swing MPP ^e^-L		+	+	+	+	+	+	+		+		+
Hang MDP ^f^-L		+	+	+	+	+	+	+		+		
Swing MDP ^f^-L		+	+	+	+	+	+	+		+		+
Hang RPP ^g^-L		+	+	+	+	+	+	+		+		
Swing RPP ^g^-L		+	+	+	+	+	+	+		+		+
Hang RDP ^h^-L		+	+	+	+	+	+	+		+		
Swing RDP ^h^-L		+	+	+	+	+	+	+		+	+	
Hang LF ^i^-L		+	+	+	+	+	+	+		+		
Swing LF ^i^-L		+	+	+	+	+	+	+		+		

*Note:*^a^ P = Palm; ^b^ T = Thumb; ^c^ IPP = Index Finger Proximal Phalanx; ^d^ IDP = Index Finger Distal Phalanges; ^e^ MPP = Medial Finger Proximal Phalanx; ^f^ MDP = Medial Finger Distal Phalanges; ^g^ RPP = Ring Finger Proximal Phalanx; ^h^ RDP = Ring Finger Distal Phalanges; ^i^ LF = Little Finger.

**Table 4:** Differences between the Hang and Swing time series (where + indicates significant difference by *t*-test (*p* < 0.05)) for left hand.

Variables	−30/s	0/s	30/s	60/s	90/s	120/s	150/s	180/s	210/s	240/s	270/s	300/s
P ^a^-L		+						+		+	+	+
T ^b^-L										+	+	+
IPP ^c^-L				+	+	+	+	+	+	+	+	+
IDP ^d^-L		+					+	+	+	+	+	+
MPP ^e^-L			+	+			+	+	+	+	+	+
MDP ^f^-L		+								+	+	+
RPP ^g^-L			+					+		+	+	+
RDP ^h^-L		+						+	+	+	+	+
LF ^i^-L		+	+			+	+	+	+	+	+	+

*Note:*^a^ P = Palm; ^b^ T = Thumb; ^c^ IPP = Index Finger Proximal Phalanx; ^d^ IDP = Index Finger Distal Phalanges; ^e^ MPP = Medial Finger Proximal Phalanx; ^f^ MDP = Medial Finger Distal Phalanges; ^g^ RPP = Ring Finger Proximal Phalanx; ^h^ RDP = Ring Finger Distal Phalanges; ^i^ LF = Little Finger.

## Data Availability

The data presented in this study are available on request from the corresponding author.
